# Hepatocyte growth factor prevents lupus nephritis in a murine lupus model of chronic graft-versus-host disease

**DOI:** 10.1186/ar2012

**Published:** 2006-07-19

**Authors:** Takanori Kuroiwa, Tsuyoshi Iwasaki, Takehito Imado, Masahiro Sekiguchi, Jiro Fujimoto, Hajime Sano

**Affiliations:** 1 Division of Rheumatology and Clinical Immunology, Department of Internal Medicine, Hyogo College of Medicine, 1-1 Mukogawa-cho, Nishinomiya, Hyogo 663-8501, Japan; 2 Division of Hematology, Department of Internal Medicine, Hyogo College of Medicine, 1-1 Mukogawa-cho, Nishinomiya, Hyogo 663-8501, Japan; 3 First Department of Surgery, Hyogo College of Medicine, 1-1 Mukogawa-cho, Nishinomiya, Hyogo 663-8501, Japan

## Abstract

Chronic graft-versus-host disease (GVHD) induced in (C57BL/6 × DBA/2) F1 (BDF1) mice by the injection of DBA/2 mouse spleen cells represents histopathological changes associated with systemic lupus erythematosus (SLE), primary biliary cirrhosis (PBC) and Sjogren's syndrome (SS), as indicated by glomerulonephritis, lymphocyte infiltration into the periportal area of the liver and salivary glands. We determined the therapeutic effect of hepatocyte growth factor (HGF) gene transfection on lupus using this chronic GVHD model. Chronic GVHD mice were injected in the gluteal muscle with either HVJ liposomes containing 8 μg of the human HGF expression vector (HGF-HVJ liposomes) or mock vector (untreated control). Gene transfer was repeated at 2-week intervals during 12 weeks. HGF gene transfection effectively prevented the proteinuria and histopathological changes associated with glomerulonephritis. While liver and salivary gland sections from untreated GVHD mice showed prominent PBC- and SS-like changes, HGF gene transfection reduced these histopathological changes. HGF gene transfection greatly reduced the number of splenic B cells, host B cell major histocompatibility complex class II expression, and serum levels of IgG and anti-DNA antibodies. IL-4 mRNA expression in the spleen, liver, and kidneys was significantly decreased by HGF gene transfection. CD28 expression on DBA/2 CD4+ T cells was decreased by the addition of recombinant HGF *in vitro*. Furthermore, IL-4 production by DBA/2 CD4+ T cells stimulated by irradiated BDF1 dendritic cells was significantly inhibited by the addition of recombinant HGF *in vitro*. These results suggest that HGF gene transfection inhibited T helper 2 immune responses and reduced lupus nephritis, autoimmune sialoadenitis, and cholangitis in chronic GVHD mice. HGF may represent a novel strategy for the treatment of SLE, SS and PBC.

## Introduction

Pathogenic T cells that recognize self-antigens and drive B cell hyperactivity play a central role in the pathogenesis of both human and murine lupus [1-3]. Chronic graft-versus-host disease (GVHD), which is induced in (C57BL/6 × DBA/2) F1 (BDF1) mice by injection of DBA/2 spleen cells, is associated with the activation of donor CD4+ T cells that recognize host major histocompatibility complex (MHC) antigens and drive host B cell hyperactivity [4,5]. Mice of this parent-into-F1 chronic GVHD model show increased T helper (Th) 2 immune responses, and exhibit autoimmune disorders that resemble human systemic lupus erythematosus, primary biliary chirrhosis, and Sjogren's syndrome, which are characterized by lymphocyte infiltration into organs such as the kidneys, liver and salivary glands [6].

In contrast, the parent-into-F1 acute GVHD model, which is induced in BDF1 mice by the injection of C57BL/6 (B6) spleen cells, is associated with the activation of donor CD8+ cytotoxic T lymphocytes (CTLs) that recognize host MHC antigens and cause death by affecting host immune and hematopoietic systems [7-9]. As acute GVHD can be inhibited by the addition of neutralizing anti-IL-2 monoclonal antibodies (mAbs) and is not induced by perforin-deficient donor T cells [10,11], acute GVHD is associated with increased Th1-mediated immune responses and with perforin expression on donor T cells.

One of the principal distinctions between the acute and chronic GVHD models appears to be the nine-fold reduction in CTL precursor numbers with anti-host specificity (which eliminate autoreactive host B cells) generated during GVHD induced by DBA/2 mouse spleen cells rather than by B6 mouse spleen cells [12]. Previous experiments have demonstrated that cytokines such as IL-12 and IL-18 induce donor anti-host CTLs in chronic GVHD mice and can ameliorate chronic GVHD, or even stimulate the development of acute GVHD [13,14].

Recently, we demonstrated that repeated transfection of skeletal muscle with the gene encoding the human hepatocyte growth factor (HGF) induced continuous production of HGF, which strongly inhibited both acute and chronic GVHD in bone marrow transplantation model mice. HGF gene transfection inhibited end-organ damage caused by acute GVHD through HGF's anti-apoptotic and regenerative actions. HGF also exerted a potent protective effect on thymus, which in turn inhibited the development of autoreactive T cells in the thymus damaged by acute GVHD [15,16]. Considering these results, HGF seems to not only inhibit end-organ damage through its anti-apoptotic and regenerative actions but also directly control autoimmunity in chronic GVHD mice. In the present study, we evaluated the therapeutic and preventive effects of HGF treatment using the parent-into-F1 chronic GVHD mouse model. Our results indicate that HGF gene transfection effectively prevented proteinuria and lymphocyte infiltration of the kidneys, liver, and salivary glands. HGF gene transfection also inhibited an increase in splenic B cell numbers, MHC class II expression by host B cells, and serum IgG and anti-DNA antibody concentrations in chronic GVHD mice. Lastly, HGF transfection inhibited IL-4 mRNA expression in the kidneys, liver, and spleen of chronic GVHD mice. Therefore, HGF may represent a novel strategy for the treatment of systemic lupus erythematosus, primary biliary cirrhosis, and Sjogren's syndrome.

## Materials and methods

### Animals

Female B6 (H-2^b^), DBA/2 (H-2^d^), and BDF1 (H-2^bxd^) mice at 8 to 12 weeks old were purchased from the Shizuoka Laboratory Animal Center (Shizuoka, Japan). All mice were maintained in a pathogen-free facility at the Hyogo College of Medicine. Animal experiments were done in accordance with the guidelines of the National Institutes of Health, as specified by the animal care policy of Hyogo College of Medicine.

### Induction of GVHD

DBA/2 mouse spleen cells (9 × 10^7^) or B6 mouse spleen cells (5 × 10^7^) were injected into non-irradiated BDF1 mice via the tail vein as previously described [7-9]. Control mice were injected with normal BDF1 mouse spleen cells (9 × 10^7^).

### Expression vector and preparation of liposomes containing hemagglutinating virus of Japan

Human HGF cDNA (2.2 kb) was inserted into the *Eco*RI and *Not*I sites in the pUC-SRα plasmid under the control of the SRα promoter [17]. Hemagglutinating virus of Japan (HVJ) liposomes containing plasmid DNA and high mobility group 1 protein were constructed as previously described [15]. Briefly, phosphatidylserine, phosphatidylcholine, and cholesterol were mixed at a ratio of 1:4.8:2 (w/w/w), and 1 mg of this lipid mixture added to 20 to 40 μg of plasmid DNA that had been complexed with 6 to 12 μg high mobility group 1 nonhistone chromosomal protein purified from calf thymus. This mixture was sonicated to form liposomes and then mixed with ultraviolet-irradiated HVJ. Excess free virus was then removed from the HVJ liposomes by sucrose density gradient centrifugation.

### Gene transfer

BDF1 mice were injected with either HVJ liposomes containing 8 μg human HGF expression vector (HGF-HVJ liposomes) or mock vector (GVHD control) into the gluteal muscle. Gene transfer was repeated at 2-week intervals from the first day of GVHD induction for 12 weeks.

### Histopathology

Tissues were fixed in 10% buffered formalin and embedded in paraffin. Sections were stained with hematoxylin and eosin and examined by light microscopy.

### Flow cytometry

Cell suspensions were prepared in PBS containing 1% fetal calf serum and 0.1% (W/V) sodium azide. Cells were incubated with anti-Fc receptor mAb (2.4G2) for 10 minutes at 4°C, and then incubated with FITC-conjugated mAb and phycoerythrin-conjugated mAb for 30 minutes. Stained cells were washed twice, resuspended, and analyzed using a FACScan (Becton Dickinson, Mountain View, California, USA). Anti-Fc receptor (2.4G2) mAb, FITC-conjugated anti-mouse H-2K^b ^(clone AF6-88.5) mAb, anti-B220 (clone RA3-6B2) mAb, anti-CD80 (B7-1) (clone 1G10) mAb, anti-CD86 (B7-2) (clone GL1) mAb, anti-CD28 (clone 37.51), and phycoerythrin-conjugated anti-mouse H-2K^d ^(clone SF1-1.1) mAb, and anti-I-A^b ^mAb (clone AF6-120.1) were all purchased from PharMingen (SanDiego, California, USA). Multicolor flow cytometry was performed as described previously, with some modifications [15,16]. Channel numbers for data integration were chosen according to the staining patterns of normal spleen cells. Staining of normal F1 spleen cells with anti-MHC antibodies gave a unimodal positive profile when compared to negative controls. Donor cells in GVHD mice were identified as subpopulations that were clearly negative for F1-specific MHC markers.

### ELISA for IL-4 and IFN-γ

Murine IL-4 and IFN-γ levels in culture supernatants were measured by ELISA using anti-mouse IL-4 and IFN-γ mAb (Genzyme Pharmaceuticals, Cambridge, Massachusetts, USA) according to the manufacturer's protocols.

### Urine protein measurement

Proteinuria was assessed semiquantitatively using urine dip sticks (Albustix; Bayer Diagnostics, Basingstoke, United Kingdom).

### Measurement of serum IgG1 and anti-ssDNA antibodies

Sera were collected from individual mice and serum levels of IgG1 and anti-single-stranded (ss)DNA antibodies determined by ELISA. Briefly, purified rat anti-mouse IgG1 mAb (A85-3; PharMingen) and HRP-conjugated rat anti-mouse IgG1 (Zymed, San Francisco, California, USA) were used for plate coating and secondary Abs, respectively, with mouse IgG1 (S1-68.1; PharMingen) used as the protein standard. Serum levels of anti-ssDNA IgG were determined by ELISA as described previously [18]. Briefly, microtiter plates were coated with heat-denatured calf thymus DNA (Sigma, St Louis, Missouri, USA), blocked with 2% BSA-PBS, and incubated with two-fold serial dilutions of experimental mouse sera beginning at a dilution of 1/50. Plates were then incubated with HRP-labeled anti-mouse IgG (Zymed) and OD measured at 405 nm.

### Mixed lymphocyte reaction and *in vitro *cytokine production

CD4+ T cells and CD11c+ dendritic cells (DCs) were purified from spleen cells by positive selection using immunomagnetic beads (Miltenyi Biotec, Auburn, California, USA). Purity of the CD4+ and CD11c+ populations was >90% and >95%, respectively. CD4+ T cells from DBA/2 (H-2^d^) mice (4 × 10^6^/ml/well) were cultured with irradiated (20 Gy) CD11c+ DCs from BDF1 (H-2^b×d^) mice (1 × 10^6^/ml/well) in 24-well flat-bottomed plates (Falcon Labware, Lincoln Park, New Jersey, USA). After 72 h, viable cells (1 × 10^5^/200 μl/well) were stimulated in 96-well flat-bottomed plates (Falcon Labware) coated with 5 μg/ml anti-CD3 mAb (PharMingen). After 48 h, IL-4 and IFN-γ concentrations in the culture supernatants were measured by ELISA.

### ^51^Cr release assay

Anti-host CTL activity was tested using spleen cells from chronic GVHD mice at 2 weeks after GVHD induction. Spleen cells (5 × 10^6^/ml/well) were restimulated with irradiated (20 Gy) BDF1 spleen cells (3 × 10^6^/ml/well) for 5 days. Effector cells were harvested and CTL activity assessed based on the lysis of EL-4 (H-2^b^) cells in a 4 h ^51^Cr release assay. Anti-host CTL activity was also tested using spleen cells from acute GVHD mice based on P815 cell (H-2^d^) lysis. Effector cells were tested in triplicate at four effector:target ratios and the percent lysis calculated according to the following formula: [(sample cpm - spontaneous cpm)/(maximum cpm - spontaneous cpm)] × 100%. Results were calculated as the mean percent lysis ± standard deviation at a given effector:target ratio for each treatment group.

### Reverse transcribed PCR

RNA was extracted using an ISOGEN (Nippongene, Toyama, Japan) according to the manufacturer's instructions, and cDNA prepared using 2.5 μM random hexamers (Applied Biosystems Inc., Foster City, California, USA). IFN-γ and IL-4 mRNA levels were quantified by real-time reverse transcribed (RT)-PCR in a total volume of 25 μL for 40 to 50 cycles of 15 s at 95°C and 1 minute at 60°C. Samples were run in triplicate, and relative expression levels determined by normalizing expression according to β-actin expression. Primer sequences used were: IFN-γ, TGGCTGTTTCTGGCTGTTACTG and AATCAGCAGCGACTCCTTTTCC; IL-4, CCAGCTAGTTGTCATCCTGCTCTTCTTTCTCG and CAGTGATGAGGACTTGGACTCATTCATGGTGC; β-actin, TGTGATGGTGGGAATGGGTCAG and TTTGATGTCACGCACGATTTCC.

### Statistical analysis

Group mean values were compared using the two-tailed Student's *t*-test. A *p *value of less than 0.05 was considered statistically significant.

## Results

### HGF reduces histopathological changes caused by chronic GVHD

**Figure 1:**
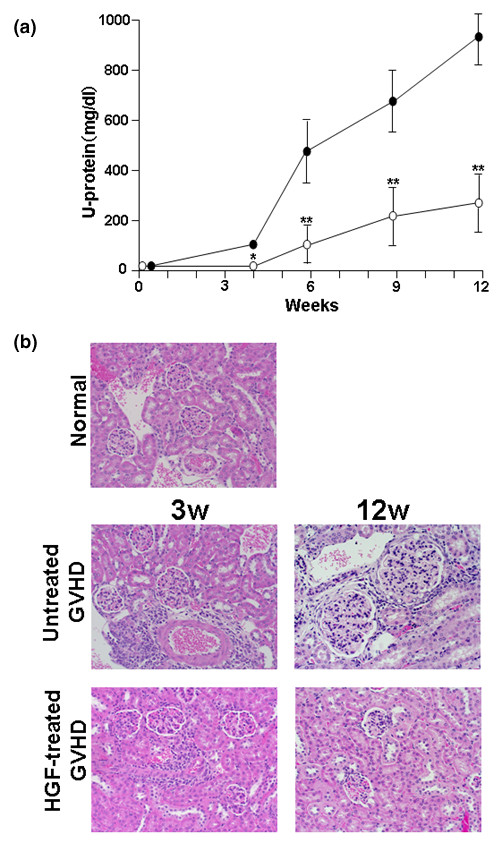
Therapeutic effects of hepatocyte growth factor (HGF) on the progression of lupus nephritis. **(a) **Protective effect of HGF against the development of nephritic syndrome, as determined by changes in urine protein excretion levels. Open circles, HGF-treated graft-versus-host disease (GVHD; *n *= 10); closed circles, untreated GVHD (*n *= 10). Data represent mean ± standard error (*n *= 10). *p < 0.05; **p < 0.01 compared to untreated GVHD at the same time point. **(b) **Histopathology of kidney tissue from untreated and HGF-treated GVHD mice. Mononuclear cell infiltration into the perivascular area of the kidney was observed at 3 weeks, and glomerulonephritis, as shown by glomerular enlargement, increased glomerular lobularity, mesangial hypercellularity, and membrane thickening was observed at 12 weeks after GVHD induction. HGF treatment significantly inhibited these pathological changes. Original magnification ×200.

**Figure 2:**
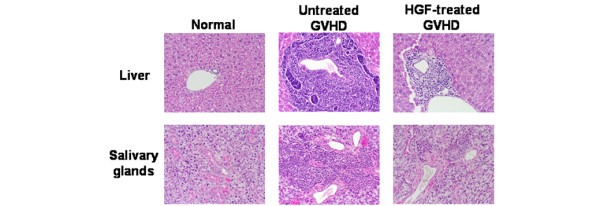
Inhibitory effect of hepatocyte growth factor (HGF) on mononuclear infiltration in the liver and the salivary glands. Chronic graft-versus-host disease (GVHD) mice were treated as described in Figure 1, and histopathology of the liver and salivary glands examined 12 weeks after GVHD induction. HGF treatment inhibited mononuclear cell infiltration into the liver and salivary glands of chronic GVHD mice. Original magnification ×200.

We investigated whether HGF gene transfection could prevent murine lupus in a model of chronic GVHD using non-irradiated parent spleen cells injected into F1 recipient mice. Increased proteinuria was observed in untreated chronic GVHD mice at 6 weeks after GVHD induction and, by 12 weeks, 6 of 10 chronic GVHD mice had features consistent with nephritic syndrome. Light microscopy analysis of kidney tissues revealed mononuclear cell infiltration into the perivascular area at 3 weeks and glomerulonephritis, as shown by glomerular enlargement, increased glomerular lobularity, mesangial hypercellularity, and increased membrane thickness, at 12 weeks. Repeated transfection of the human HGF gene into skeletal muscle of chronic GVHD mice achieved a plasma HGF level (human and mouse HGF) between 1.07 and 1.35 ng/ml during the 12 week period after GVHD induction. HGF gene transfection significantly inhibited proteinuria (Figure 1a) and histopathological changes associated with chronic GVHD (Figure 1b). HGF treatment also inhibited mononuclear cell infiltration into the liver and salivary glands of chronic GVHD mice (Figure 2).

### HGF reduces splenic B cell numbers and serum IgG1 and anti-DNA antibody concentrations in chronic GVHD mice

**Figure 3:**
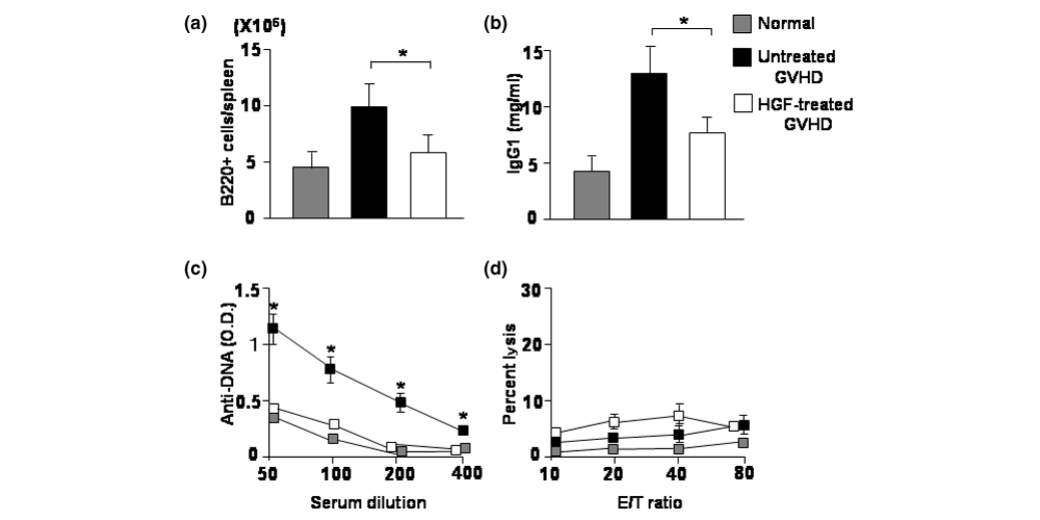
Effect of hepatocyte growth factor (HGF) on host B cell activation and donor anti-host cytotoxic T lymphocyte (CTL) activity. Chronic graft-versus-host disease (GVHD) mice were treated as described in Figure 1. **(a) **At 2 weeks after GVHD induction, spleens were removed and H-2K^b^+B220+ cells examined by flow cytometry. Data represent mean ± SD for four mice per group. **(b) **At 2 weeks after GVHD induction, serum IgG1 concentrations were determined by ELISA. Data represent mean ± SD for four mice per group. **(c) **At 2 weeks after GVHD induction, serum anti-single-stranded DNA antibody concentrations were determined by ELISA. Data represent mean ± SD for four mice per group. **(d) **At 2 weeks after GVHD induction, spleen cells were stimulated with irradiated BDF1 spleen cells for 5 days. Cytotoxicity was determined by CTL activity against ^51^Cr-labeled EL-4 (H-2^b^) target cells. CTL activity against P815 cells (H-2^d^) by acute GVHD mouse spleen cells was used as a positive control. Effector cells were tested in triplicate at four effector:target (E/T) ratios and the percent lysis calculated. Data represent mean ± SD for four mice per group.

The number of host B cells decreased by 35% in HGF gene transfected chronic GVHD mice compared to untreated chronic GVHD mice (Figure 3a). Furthermore, serum IgG1 and anti-ssDNA antibody concentrations were significantly decreased by HGF gene transfection (Figure 3b,c). Several investigators have previously shown that treatment with IL-12 or IL-18 induced donor anti-host CTLs that eliminated host B cells in chronic GVHD mice [13,14]. Therefore, we investigated whether HGF gene transfection induced donor anti-host CTLs in our chronic GVHD mouse model. To examine donor anti-host CTLs, spleen cells harvested 14 days after GVHD induction were stimulated with 20 Gy irradiated BDF1 spleen cells for 5 days, and then the cytotoxic activity of the cultured cells analyzed using ^51^Cr-labeled EL-4 (H-2^b^) or P815 (H-2^d^) as target cells. While spleen cells from both untreated and HGF-treated chronic GVHD mice showed no specific CTL activity toward host-type EL-4 cells (Figure 3d), spleen cells from acute GVHD mice showed CTL activity against host-type P815 cells (data not shown). This result indicated that HGF gene transfection did not appear to induce donor anti-host CTLs, which might explain the elimination of activated host B cells in treated chronic GVHD mice.

### HGF reduces IL-4 mRNA expression in target organs of chronic GVHD

**Figure 4:**
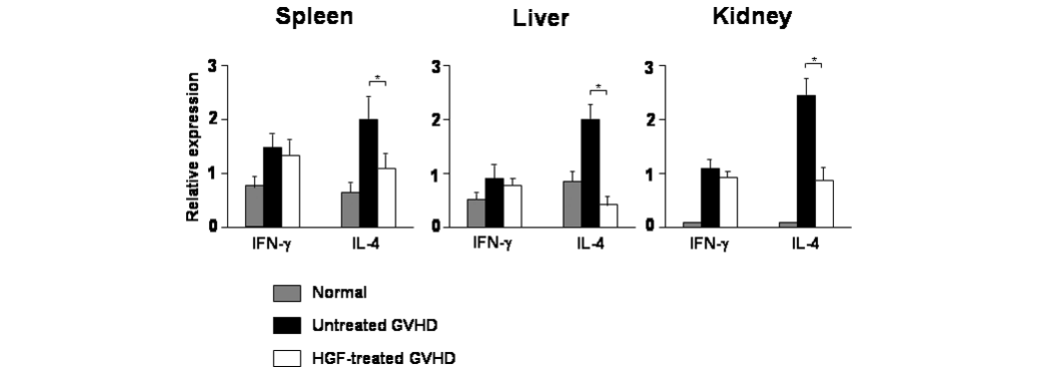
Effect of hepatocyte growth factor (HGF) on cytokine gene expression by chronic graft-versus-host disease (GVHD) mice. Chronic GVHD mice were treated as described in Figure 1. At 2 weeks after GVHD induction, spleen, liver, and kidneys were removed and IFN-γ and IL-4 mRNA expression determined by RT-PCR analysis. Data represent mean ± SD for four mice per group. *p < 0.05 compared to untreated GVHD.

Injection of DBA/2 spleen cells into BDF1 mice resulted in Th2 activation, as shown by the elevated IL-4 mRNA levels in target organs of mice with chronic GVHD [19,20]. Chronic GVHD is inhibited by the injection of anti-IL-4 mAb, which suggests a critical role for IL-4 in this disease [21,22]. We examined the effect of HGF gene transfection on IL-4 and IFN-γ mRNA expression levels in target chronic GVHD organs in the mouse. Expression of both IL-4 and IFN-γ mRNA was increased in the kidneys, liver, and spleen of untreated chronic GVHD mice 2 weeks after GVHD induction. HGF gene transfection significantly inhibited the expression of IL-4 mRNA in these organs, while IFN-γ mRNA expression levels were not significantly altered between HGF-treated and untreated chronic GVHD mice (Figure 4).

### HGF reduces MHC class II expression on host B cells

**Figure 5:**
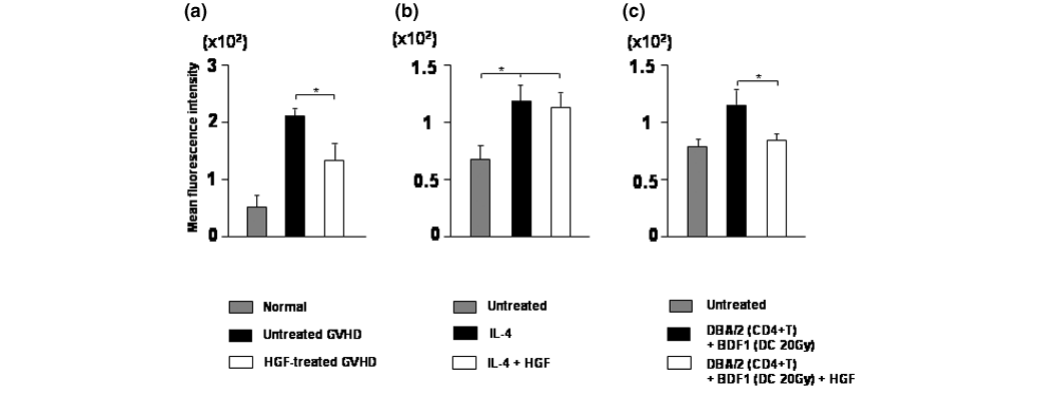
Effect of hepatocyte growth factor (HGF) on B cell MHC class II expression. **(a) **Chronic graft-versus-host disease (GVHD) mice were treated as described in Figure 1. At 2 weeks after GVHD induction, spleens were removed and the mean fluorescence intensity of MHC class II (I-A^b^) expression on B220+ cells determined. Data represent mean ± SD for four mice per group. *p < 0.05. **(b) **B220+ cells from BDF1 mice (2 × 10^6^/ml) were cultured with mouse recombinant IL-4 (10 ng/ml) in the presence or absence of human recombinant HGF (10 ng/ml) for 48 h, and mean fluorescence intensity of MHC class II (I-A^b^) expression on B220+ cells determined. Data represent mean ± SD of three independent experiments. *p < 0.05. **(c) **CD4+ T cells from DBA/2 (H-2^d^) mice (4 × 10^6^/ml/well) were cultured with irradiated (20 Gy) CD11c+ dendritic cell (DCs) from BDF1 (H-2^b×d^) mice (1 × 10^6^/ml/well) in the presence or absence of human recombinant HGF (10 ng/ml). After 72 h, viable cells (1 × 10^6^/ml) were cultured with B220+ cells from BDF1 mice (2 × 10^6^/ml) for 48 h and mean fluorescence intensity of MHC class II (I-A^b^) expression on B220+ cells determined. Data represent mean ± SD of three independent experiments. *p < 0.05.

Chronic GVHD requires continuous donor CD4+ T cell activation through recognition of host MHC class II antigens. This results in the secretion of predominantly Th2 cytokines and the stimulation of autoreactive B cells that differentiate into autoantibody-secreting cells [4-6]. It is possible that HGF may decrease the expression of MHC class II antigens on host B cells, which would suppress donor CD4+ T cell activation, and so reduce the Th2 response in chronic GVHD mice. Therefore, we examined the effect of HGF on host B cell MHC class II expression in our chronic GVHD mouse model. While untreated chronic GVHD mice expressed high levels of MHC class II antigens on host B cells, host B cells from HGF-treated chronic GVHD mice showed reduced expression (Figure 5a). To determine whether this reduced B cell MHC class II expression was a direct effect of HGF, we examined the effect of HGF on IL-4-induced MHC class II expression by B cells *in vitro*. BDF1 B cells were cultured in the presence of IL-4 with or without HGF, and MHC class II expression examined after 2 days of culture. Addition of HGF did not affect B cell MHC class II expression (Figure 5b). Another possibility is that donor DBA/2 CD4+ T cells differentiate into Th2 in response to host MHC antigens and induce MHC class II expression on host B cells in chronic GVHD mice. Therefore, we examined the ability of cultured DBA/2 anti-BDF1 MLR cells to induce MHC class II expression on BDF1 B cells. DBA/2 CD4+ T cells were cultured with 20 Gy irradiated BDF1 DCs in the presence or absence of HGF for 3 days, after which viable cells were cultured with BDF1 B cells and MHC class II expression assessed. While MHC class II expression by BDF1 B cells was significantly increased in the presence of viable MLR cells in the absence of HGF, expression levels were not increased in the presence HGF (Figure 5b). Thus, our results indicated that HGF may inhibit the ability of donor DBA/2 T cells to induce MHC class II expression by host BDF1 B cells in chronic GVHD mice.

### *In vitro *treatment with HGF reduces Th2 generation from DBA/2 CD4+ T cells

**Figure 6:**
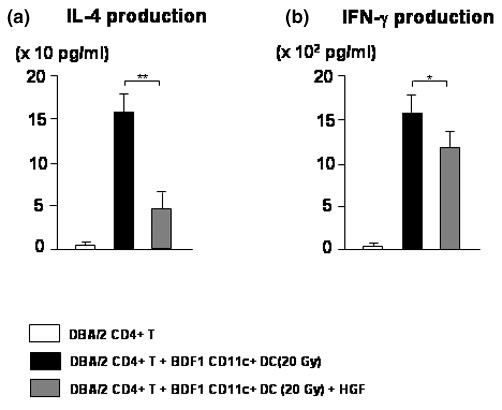
*In vitro *effect of hepatocyte growth factor (HGF) on T helper (Th)2 generation from DBA/2 CD4+ T cells. CD4+ T cells from DBA/2 (H-2^d^) mice (4 × 10^6^/ml/well) were cultured with irradiated (20 Gy) CD11c+ dendritic cell (DCs) from BDF1 (H-2^b×d^) mice (1 × 10^6^/ml/well) in the presence or absence of human recombinant HGF (10 ng/ml). CD4+ T cells from DBA/2 (H-2^d^) mice (4 × 10^6^/ml/well) cultured without CD11c+ DC stimulation were used as controls. After 72 h, viable cells (1 × 10^5^/200 μl/well) were harvested and stimulated with anti-CD3 mAb (5 μg/ml) for 48 h. The concentration of IL-4 (a) and IFN-γ (b) in the culture supernatant was measured by ELISA. Data represent mean ± SD of three independent experiments. *p < 0.05; **p < 0.01.

As donor DBA/2 CD4+ T cells differentiate into Th2 cells in response to host BDF1 antigen presenting cells in chronic GVHD mice, we performed a DBA/2 anti-BDF1 MLR and examined the effect of HGF on the generation of Th1 and Th2. Responder CD4+ T cells from DBA/2 mice were cultured with 20 Gy irradiated CD11c+ DCs from BDF1 mice with or without HGF. After 3 days culture, viable cells were stimulated by culture with anti-CD3 mAb for 48 h, and IL-4 and IFN-γ levels in the culture supernatant assayed by ELISA. While both IL-4 and IFN-γ production was inhibited by HGF, the inhibitory effect of HGF was greater on IL-4 production than on IFN-γ production (Figure 6). This suggested that HGF inhibited Th2 generation from DBA/2 CD4+ T cells stimulated by BDF1 DCs.

### *In vitro *treatment with HGF down-regulates CD28 expression on CD4+ T cells

**Figure 7:**
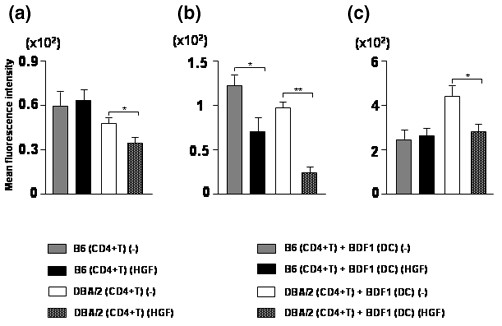
Effect of hepatocyte growth factor (HGF) on CD4+ T cell CD28 expression and on CD11c+ dendritic cell (DC) CD86 expression. **(a) **CD4+ T cells from DBA/2 (H-2^d^) or B6 (H-2^b^) mice (4 × 10^6^/ml/well) were cultured in the presence or absence of human recombinant HGF (10 ng/ml). After 48 h, mean fluorescence intensity of CD28 expression on CD4+ T cells determined. Data represent mean ± SD of three independent experiments. *p < 0.05. **(b,c) **CD4+ T cells from DBA/2 (H-2^d^) or B6 (H-2^b^) mice (4 × 10^6^/ml/well) were cultured with irradiated (20 Gy) CD11c+ DCs from BDF1 (H-2^b×d^) mice (1 × 10^6^/ml/well) in the presence or absence of human recombinant HGF (10 ng/ml) for 48 h, and mean fluorescence intensity of CD28 expression on CD4+ T cells (b) and CD86 expression on CD11c+ DCs (c) determined. Data represent mean ± SD of three independent experiments. *p < 0.05; **p < 0.01.

To investigate mechanisms of HGF to reduce Th2 generation from DBA/2 CD4+ T cells, we examined the effect of HGF on costimulatory molecules on CD4+ T cells and DCs. Previous studies have shown that the development of Th2 responses depends on CD86 interaction with CD28 [23,24]. We examined the effect of recombinant HGF on CD28 expression on CD4+ T cells and CD86 expression on CD11c+ DCs. DBA/2 or B6 CD4+ T cells were cultured in the presence or absence of recombinant HGF, and CD28 expression on CD4+ T cells was examined. Addition of HGF down-regulated CD28 expression on DBA/2 CD4+ T cells but not on B6 CD4+ T cells (Figure 7a). We next performed a DBA/2 or B6 anti-BDF1 MLR for 48 h and examined the effect of recombinant HGF on CD28 expression on CD4+ T cells. CD28 expression on CD4+ T cells from both DBA/2 and B6 mice was down-regulated by the addition of recombinant HGF, but this down-regulating activity of HGF was stronger in DBA/2 CD4+ T cells than in B6 CD4+ T cells (Figure 7b). CD86 expression on BDF1 DCs cultured with DBA/2 CD4+ T cells was also down-regulated by the addition of recombinant HGF (Figure 7c). These results suggest that down-regulation of CD86 interaction with CD28 by HGF inhibits subsequent Th2 differentiation of DBA/2 CD4+ T cells.

## Discussion

The present study demonstrates that repeated HGF gene transfection into BDF1 mice prevents the development of chronic GVHD induced by the injection of DBA/2 spleen cells. HGF transfection significantly inhibited proteinuria and histopathological changes of the kidneys, liver and salivary glands caused by chronic GVHD.

The parent-to-F1 murine model of chronic GVHD exhibits Th2-mediated immune responses, such as polyclonal B cell activation, autoantibody formation, and decreased CTL responses, that closely resemble lupus-like autoimmune disease. Rus and colleagues [5] reported that Th2 cytokine secretion and B cell activation may be early events in both acute and chronic GVHD. It is thought that the transition to chronic GVHD involves the failure of CD8+ anti-host CTLs to kill activated host B cells [5]. In this context, therapeutic strategies tested in chronic GVHD mice have included the use of Th1-inducing cytokines [13,14]. Early treatment with IL-12 (on days 0 to 4 after induction of chronic GVHD) but not late treatment (on days 8 to 12 after induction of chronic GVHD) generated anti-host CTLs that eliminated host autoantibody-producing B cells, thereby converting chronic GVHD into acute GVHD [13]. However, regardless of treatment schedule (treatment on days 0 to 5 or days 8 to 13 after induction of chronic GVHD), IL-18 treatment generated anti-host CTLs, but did not induce acute GVHD [14].

In contrast to Th1-inducing cytokine treatment, HGF did not generate anti-host CTLs *in vivo*. Although Th1-inducing cytokines induced the development of DBA/2-derived Th1 in a DBA/2 anti-BDF1 MLR [25], the addition of HGF did not induce Th1 *in vitro *(data not shown). However, HGF treatment inhibited IL-4 mRNA expression in chronic GVHD target organs, splenic B cell expansion, and autoantibody production in chronic GVHD mice. Thus, it appears that HGF inhibited lupus-like autoimmune disease through the inhibition of Th2 generation rather than by induction of Th1.

The precise mechanisms by which HGF inhibits Th2-mediated responses in chronic GVHD mice remain unclear. Possible mechanisms include that HGF suppresses MHC class II expression by host B cells, leading to reduced antigen presentation to donor CD4+ T cells. Indeed, we observed decreased MHC class II expression on host B cells from HGF-treated chronic GVHD mice. While the addition of HGF to an *in vitro *DBA/2 anti-BDF1 MLR inhibited the capacity of BDF1 B cells to increase MHC class II expression, HGF did not inhibit IL-4-induced B cell MHC class II expression, which suggested that HGF did not directly suppress MHC class II expression on host B cells.

DCs play a pivotal role in determining the balance between responsiveness and tolerance in the immune system [26,27], and the persistence of host DCs following bone marrow transplantation is correlated with the development of severe acute and chronic GVHD [28,29]. Chronic DC activation, such as by CD40L over-expression in basal epidermal layers that accelerates DC maturation, leads to autoimmunity [30]. Immature myeloid DCs are loaded with self-antigen-derived molecules and, upon interaction with T cells, deliver a signal that results in T cell deletion and anergy [31]. Modified myeloid DCs can act as regulatory DCs to protect against acute GVHD [32]. We observed that in a DBA/2 anti-BDF1 MLR, which contains DBA/2 CD4+ T cells and BDF1 DCs, the addition of HGF significantly inhibited the generation of DBA/2 Th2. We also observed that c-Met/HGF receptors are expressed by both DCs and CD4+ T cells (data not shown) and that HGF significantly down-regulated CD28 expression on DBA/2 CD4+ T cells stimulated by BDF1 DCs. We also observed down-regulation of CD86 expression on BDF1 DCs cultured with DBA/2 CD4+ T cells. HGF may act on the CD28-CD86 pathway, thereby inhibiting Th2 generation.

We recently demonstrated that repeated transfection of the human HGF gene into skeletal muscle in a bone marrow transplantation model of GVHD promoted hematopoietic function and strongly inhibited acute GVHD by limiting tissue damage and the subsequent endotoxin-mediated inflammatory cascade [15]. The principal mechanisms by which HGF blocks acute GVHD appeared to involve the protection of target organs from injury through anti-apoptotic effects and the inhibition of subsequent inflammatory cytokine reactions [16]. In the present study, we demonstrated that HGF inhibited the increased Th2-mediated immune response in chronic GVHD mice. Thus, HGF treatment may be beneficial for both Th1-mediated acute GVHD and Th2-mediated autoimmune chronic GVHD.

## Conclusion

HGF gene transfection effectively prevented the proteinuria and histopathological changes associated with glomerulonephritis, primary biliary cirrhosis and Sjogren's syndrome. HGF gene transfection greatly reduced the number of splenic B cells, host B cell MHC class II expression, and serum levels of IgG and anti-DNA antibodies. IL-4 mRNA expression in the spleen, liver, and kidneys was significantly decreased by HGF gene transfection. CD28 expression on DBA/2 CD4+ T cells and CD86 expression on BDF1 DCs was decreased by the addition of recombinant HGF *in vitro*. Furthermore, IL-4 production by DBA/2 CD4+ T cells stimulated by irradiated BDF1 DCs was significantly inhibited by the addition of recombinant HGF *in vitro*. These results suggested that HGF gene transfection inhibited Th2 immune responses and reduced lupus nephritis, autoimmune sialoadenitis, and cholangitis in chronic GVHD mice. HGF may represent a novel strategy for the treatment of systemic lupus erythematosus, Sjogren's syndrome and primary biliary cirrhosis.

## Abbreviations

CTL = cytotoxic T lymphocyte; DC = dendritic cell; ELISA = enzyme-linked immunosorbent assay; E/T – effector:target; FITC = fluorescein isothiocyanate; GVHD = graft-versus-host disease; HGF = hepatocyte growth factor; HVJ = hemagglutinating virus of Japan; IFN = interferon; IL = interleukin; mAb = monoclonal antibodies; MHC = major histocompatibility complex; MLR = mixed lymphocyte reaction; PBS = phosphate-buffered saline; RT-PCR = reverse transcribed PCR; SD = standard deviation; Th = T helper.

## Competing interests

The authors declare that they have no competing interests.

## Authors' contributions

TK, TI (Takehito Imado), and MS performed the animal study. TI (Tsuyoshi Iwasaki) conceived of the study, participated in the design and coordination of the study, and participated in the interpretation of the results. JF participated in the HGF gene preparation and transfection. HS participated in the design and interpretation of the results. Tsuyoshi Iwasaki and Takanori Kuroiwa contributed equally to this work and should be considered as joint first authors.
